# Left Ventricular Pseudoaneurysm After Sutureless Repair for Post-Myocardial Infarction Oozing-Type Left Ventricular Free Wall Rupture

**DOI:** 10.7759/cureus.71832

**Published:** 2024-10-19

**Authors:** Akihiro Sasahara, Kouki Nakashima, Yuki Tanaka, Yuta Murai, Kuniyoshi Ohara

**Affiliations:** 1 Department of Cardiovascular Surgery, Ebina General Hospital, Ebina, JPN; 2 Department of Cardiovascular Surgery, Sagamihara Kyodo Hospital, Sagamihara, JPN

**Keywords:** cardiac infarction, cardiac rupture, left ventricular pseudoaneurysm, mechanical complication, sutureless repair technique

## Abstract

A 66-year-old male patient underwent repair using the sutureless technique for an oozing-type cardiac rupture associated with myocardial infarction of the anterior left ventricular wall. On postoperative day 10, echocardiography revealed a thrombus in the apex region, and anticoagulation therapy was started with warfarin. The thrombus disappeared two months postoperatively. Four months after surgery, the patient came to the hospital with a chief complaint of breathlessness. The computer tomography scan showed a pseudoaneurysm in the left ventricular anterior wall. Because of breathlessness due to decreased effective cardiac output associated with the left ventricular mass and the risk of rupture of the left ventricular pseudoaneurysm, we decided that surgery was indicated. When the left ventricular pseudoaneurysm was incised under cardiac arrest using an artificial heart-lung machine, an oval-shaped communication orifice was found, which was closed directly with continuous sutures. The breathlessness disappeared and the patient was discharged 13 days after surgery.

## Introduction

Left ventricular free wall rupture (LVFWR) is one type of cardiac rupture, a rare but often fatal complication that can occur following acute myocardial infarction (AMI). Despite advances in medical management and early revascularization, LVFWR remains a significant cause of mortality in patients with AMI, particularly in the elderly and those with delayed reperfusion. LVFWR typically occurs within the first week after infarction, though it can also present in the subacute or chronic phases. Factors such as persistent hypertension, and delayed reperfusion therapy increase the risk of rupture [1].

LVFWR is classified into two main types, blowout and oozing. the blowout type is characterized by a sudden and fatal rupture that often occurs early after onset. In contrast, the oozing type tends to progress slowly, with symptoms gradually emerging, often accompanied by pericardial effusion or cardiac tamponade. Although less dramatic in presentation, the oozing type can also lead to fatal cardiac tamponade if not treated in a timely manner.

Surgical repair is the only life-saving treatment for both types of LVFWR, with the outcome dependent on the rupture size and location as well as the patient’s overall condition [2]. While conventional suture repair is the standard approach for surgical treatment, both conventional suture repair and sutureless techniques, such as the use of biological adhesives and patches, have been reported for the oozing type. The sutureless techniques offer promising options when there is a need to avoid the use of cardiopulmonary bypass or to minimize further damage to the myocardial tissue.

In this case report, we describe a patient who developed a left ventricular pseudoaneurysm in the chronic phase after undergoing sutureless repair of an oozing-type cardiac rupture associated with anterior wall myocardial infarction.

## Case presentation

**Figure 1:**
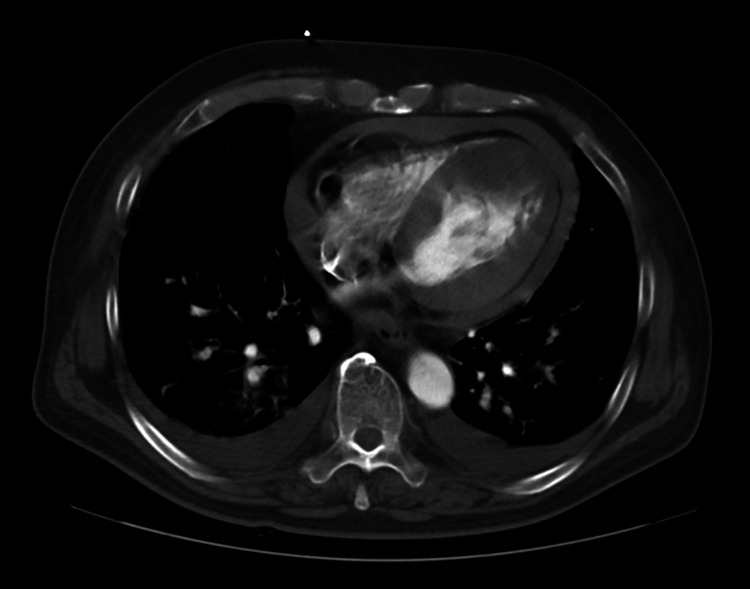
Computed tomography showing a circumferential hemopericardium

**Figure 2:**
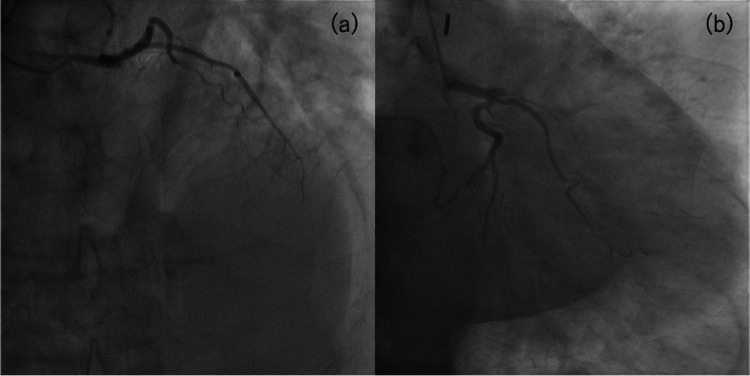
Coronary angiography showing a proximal left anterior descending artery occlusion (a) Straight cranial, (b) Right anterior oblique

**Figure 3:**
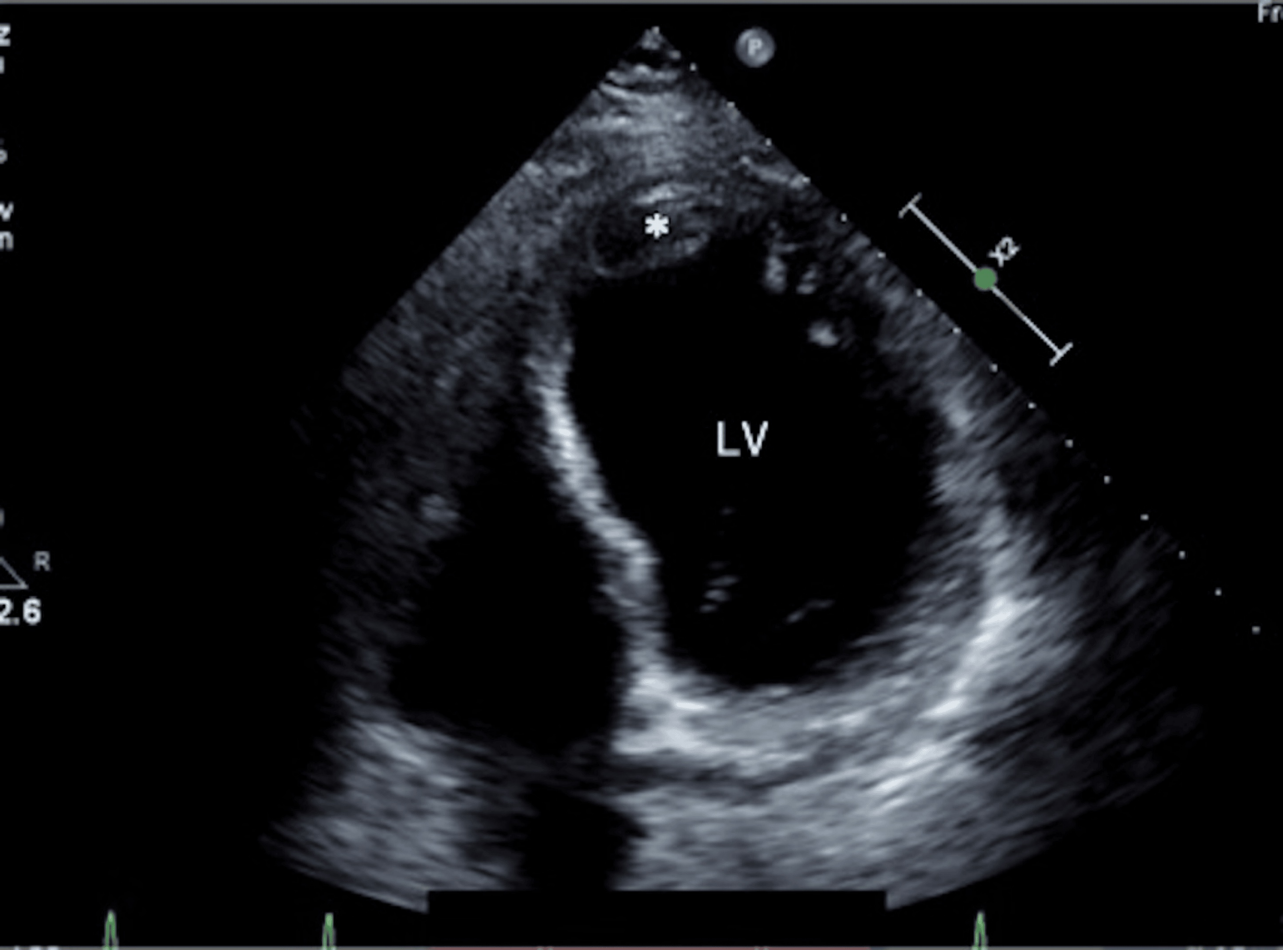
Transthoracic echocardiography showing a thrombus (*) at the left ventricle LV: left ventricle

A 66-year-old male patient with a hypertension history presented with chest pain and was urgently transported to our hospital. The electrocardiogram (ECG) revealed ST elevation in leads I, aVL, and V2-V6. Transthoracic echocardiography revealed hypokinesis of the left ventricular anterior wall and the presence of pericardial effusion. Computed tomography (CT) revealed a circumferential hemopericardium, resulting in an oozing-type LVFWR diagnosis after an acute myocardial infarction that involved the anterior wall and apex (Figure 1). Coronary angiography was performed after an intra-aortic balloon pump insertion, and it revealed a proximal left anterior descending artery occlusion (Figure 2), which required same-day emergency surgery. Intraoperatively, a myocardial injury sutureless repair was performed with TachoSil (Corza Medical, Westwood, Massachusetts, United States) that was applied to the damaged myocardium, followed by Beriplast (CSL Behring, King of Prussia, Pennsylvania, United States) and bovine pericardial patch placement. Follow-up transthoracic echocardiography on postoperative day 10 detected a thrombus at the cardiac apex (Figure 3). Anticoagulation therapy with warfarin was initiated because the thrombus was immobile. The patient was discharged on postoperative day 19 after achieving therapeutic prothrombin time-international normalized ratio levels. Echocardiography revealed apical thrombus resolution two months postoperatively. However, the patient developed shortness of breath six months postoperatively, prompting further evaluation. Transthoracic echocardiography detected a mass in the left ventricular anterior wall.

**Figure 4:**
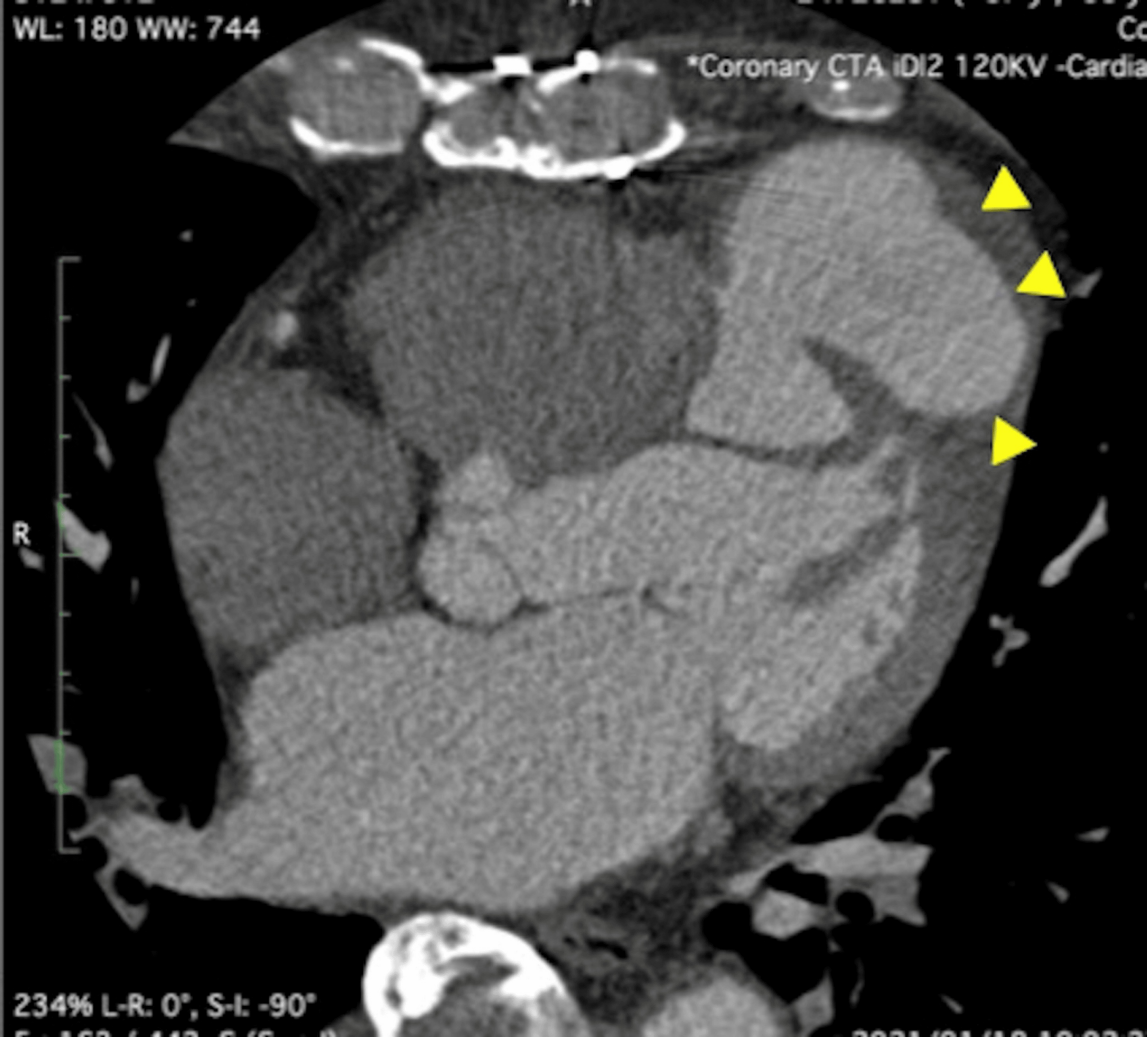
Cardiac computed tomography demonstrating pseudoaneurysm (yellow triangles) in the left ventricle

The patient’s height was 175 cm, weight was 77 kg, blood pressure was 100/60 mmHg, heart rate was 72 bpm, and blood oxygen level was 97%. Laboratory results included a hemoglobin level of 11 mg/dL, creatinine of 1.1 mg/dL, and an estimated glomerular filtration rate of 52 mL/min/1.73 m². The ECG revealed a heart rate of 72 bpm with a QS pattern in leads V2-V4. Transthoracic echocardiography demonstrated an ejection fraction of 40%, a left ventricular end-diastolic diameter of 56 mm, and a left ventricular end-systolic diameter of 40 mm. Hypokinesis was observed in the anterior wall, extending from the interventricular septum to the apex and lateral wall, with the apex exhibiting non-thinning and aneurysmal changes. Cardiac CT demonstrated a pseudoaneurysm measuring 59 × 55 × 33 mm protruding cranially from the left ventricular anterior wall, with a thrombus present within the aneurysm (Figure 4).

**Figure 5:**
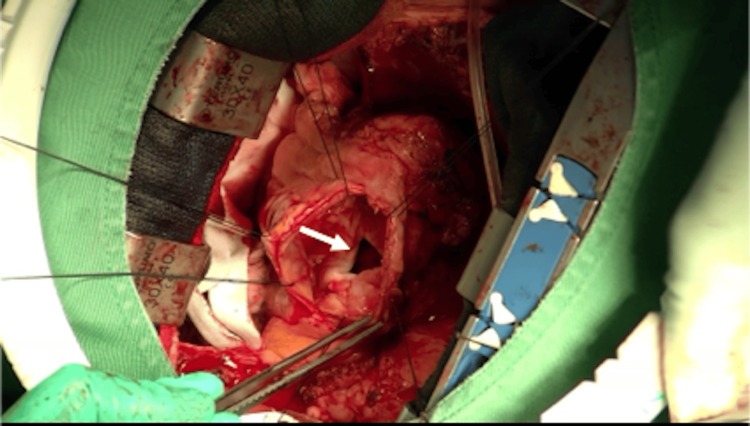
Excision of the pseudoaneurysm exposing an oval-shaped communication orifice (arrow) measuring 30 × 25 mm

**Figure 6:**
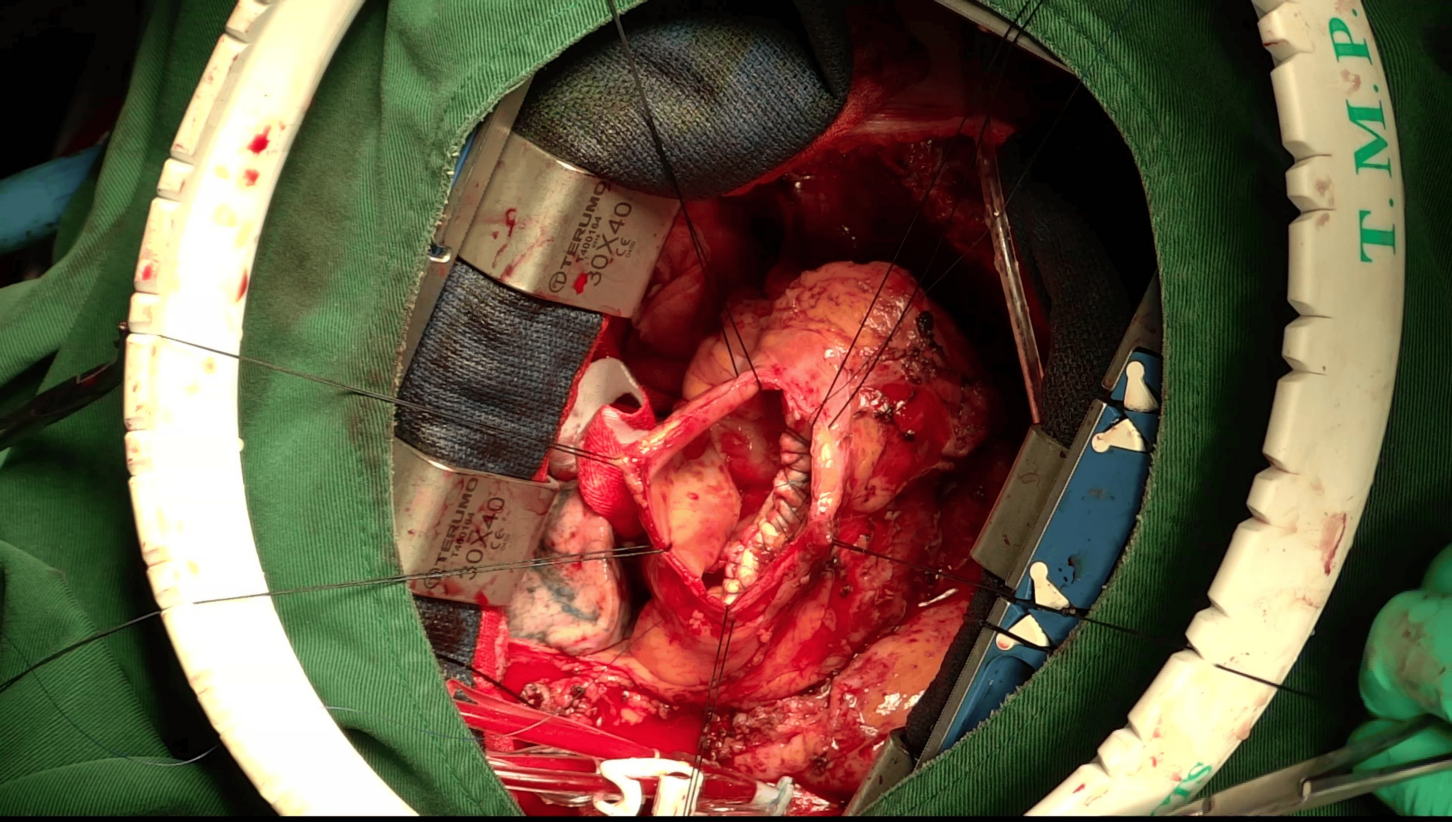
Intraoperative photograph showing closure of the defect with felt patches.

Surgery was deemed necessary, considering the patient’s shortness of breath due to reduced effective cardiac output related to ventricular aneurysm, as well as the risk of rupture of the pseudoaneurysm. The surgery involved a median re-sternotomy and dissection of adhesions while simultaneously exposing the femoral vessels for cannulation. An additional venous cannula was placed in the superior vena cava, and cardiopulmonary bypass was started. A wall demonstrating paradoxical movement was determined on the anterior surface of the left ventricle, which confirmed the presence of the pseudoaneurysm. The ascending aorta was clamped, and cardioplegia was administered antegrade to achieve cardiac arrest. The pseudoaneurysm was excised, and an oval-shaped communication orifice measuring 30 × 25 mm was exposed (Figure 5). The thrombus within the aneurysm was removed, and the defect was directly closed with continuous sutures, reinforced with felt patches on both sides (Figure 6).

**Figure 7:**
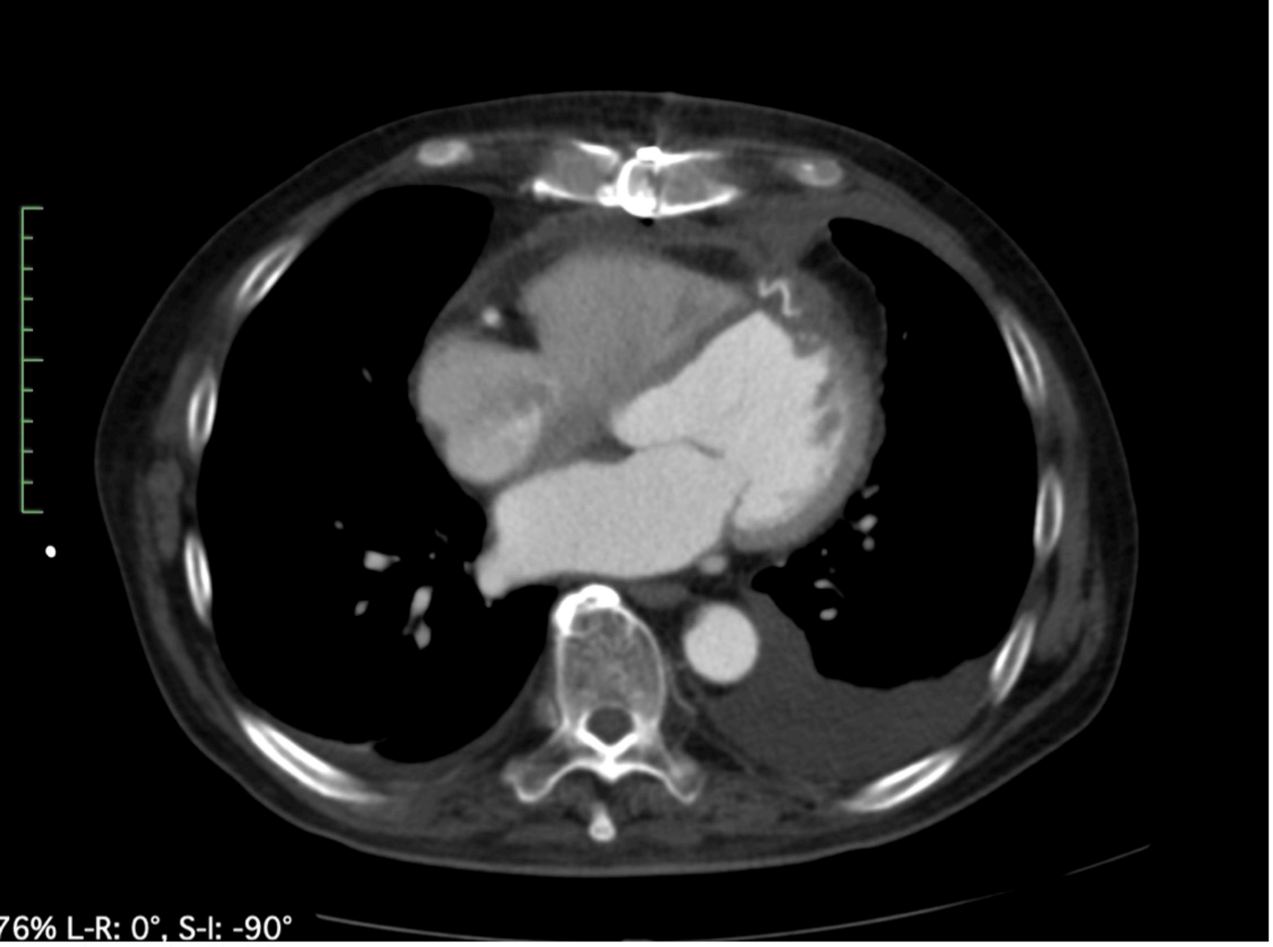
Computed tomography (seventh postoperative day) confirming the repair of the pseudoaneurysm

The patient was weaned from the ventilator two days postoperatively. Contrast-enhanced CT was conducted seven days postoperatively, and the pseudoaneurysm was confirmed to be repaired (Figure 7). The subjective symptoms of shortness of breath improved, and the patient was discharged from the hospital 13 days postoperatively.

## Discussion

Hemostasis is frequently achieved intraoperatively in cases of oozing-type cardiac rupture, and favorable outcomes have been reported with sutureless techniques without cardiopulmonary bypass [3]. However, rare reports have described left ventricular pseudoaneurysm formation after sutureless repair [4]. Thoroughly removing the epicardial hematoma and ensuring proper adhesion between the epicardium and the patch is essential to prevent pseudoaneurysm formation postoperatively [5]. Additionally, using a larger patch that extends beyond the ischemic area and covers healthy myocardium is crucial for preventing pseudoaneurysm formation [6]. 

TachoSil was applied to the damaged myocardium during the initial surgery in this case, followed by a large bovine pericardial patch. However, the patient developed a left ventricular thrombus postoperatively, requiring anticoagulation therapy with warfarin. Warfarin administration may have contributed to hematoma formation between the epicardium and the patch, thereby developing pseudoaneurysm. The pseudoaneurysm was closed directly with felt patches placed on both sides of the defect during the subsequent surgery. However, a patch is advisable to avoid excessive myocardial tension and prevent circumflex artery or coronary sinus deformation in cases where the defect is large or located near the base of the heart [7].

## Conclusions

This case illustrates the risk for pseudoaneurysm development following sutureless repair of an oozing-type left ventricular rupture. Areas with impaired wall motion are prone to thrombus formation after myocardial infarction, which may require anticoagulation therapy. However, anticoagulation increases the risk of hematoma formation between the epicardium and patch, which can result in a pseudoaneurysm. Therefore, when employing a sutureless technique to repair oozing-type cardiac rupture after acute myocardial infarction, there is a potential risk of pseudoaneurysm formation that warrants careful attention. In addition to precise intraoperative techniques, vigilant postoperative monitoring is essential.
